# Career-computer simulation increases perceived importance of learning about rare diseases

**DOI:** 10.1186/s12909-021-02688-7

**Published:** 2021-05-17

**Authors:** Babak Sarrafpour, Shwetha Hegde, Eduardo Delamare, Ruth Weeks, Rebecca A. Denham, Alix Thoeming, Hans Zoellner

**Affiliations:** 1 The Cellular and Molecular Pathology Research Unit, Oral Pathology and Oral Medicine, Discipline of Oral Surgery, Medicine and Diagnostics The School of Dentistry, The Faculty of Medicine and Health, The University of Sydney, Westmead Centre for Oral Health, Westmead Hospital, Westmead, NSW 2145 Australia; 2 Oral Radiology, Discipline of Oral Surgery, Medicine and Diagnostics The School of Dentistry, The Faculty of Medicine and Health, The University of Sydney, Sydney Dental Hospital, Surry Hills, NSW 2010 Australia; 3 Educational Innovation Team, University of Sydney, Fisher Library, Camperdown, NSW 2006 Australia; 4 Biomedical Engineering, Mechanical Engineering Building, Faculty of Engineering, Darlington Campus, The University of Sydney, Sydney, NSW 2006 Australia

**Keywords:** Rare diseases, Career experience, Computer simulation, In-class survey, Student motivation

## Abstract

**Background:**

Rare diseases may be defined as occurring in less than 1 in 2000 patients. Such conditions are, however, so numerous that up to 5.9% of the population is afflicted by a rare disease. The gambling industry attests that few people have native skill evaluating probabilities. We believe that both students and academics, under-estimate the likelihood of encountering rare diseases. This combines with pressure on curriculum time, to reduce both student interest in studying rare diseases, and academic content preparing students for clinical practice. Underestimation of rare diseases, may also contribute to unhelpful blindness to considering such conditions in the clinic.

**Methods:**

We first developed a computer simulation, modelling the number of cases of increasingly rare conditions encountered by a cohort of clinicians. The simulation captured results for each year of practice, and for each clinician throughout the entirety of their careers. Four hundred sixty-two theoretical conditions were considered, with prevalence ranging from 1 per million people through to 64.1% of the population. We then delivered a class with two in-class on-line surveys evaluating student perception of the importance of learning about rare diseases, one before and the other after an in-class real-time computer simulation. Key simulation variables were drawn from the student group, to help students project themselves into the simulation.

**Results:**

The in-class computer simulation revealed that all graduating clinicians from that class would frequently encounter rare conditions. Comparison of results of the in-class survey conducted before and after the computer simulation, revealed a significant increase in the perceived importance of learning about rare diseases (*p* < 0.005).

**Conclusions:**

The computer career simulation appeared to affect student perception. Because the computer simulation demonstrated clinicians frequently encounter patients with rare diseases, we further suggest this should be considered by academics during curriculum review and design.

**Supplementary Information:**

The online version contains supplementary material available at 10.1186/s12909-021-02688-7.

## Background

Although the problem of sufficient awareness of students and clinicians to consideration of rare diseases has been noted by others [1–9], students frequently express doubt on the value of learning about rare diseases [10]. One survey found that although 95.4% of medical students felt their knowledge about rare diseases was insufficient or very poor, 45.7% felt it was not necessary to include additional material on rare diseases in their curricula to address this [7].

Many medical educators make significant effort to raise awareness of rare diseases amongst students. However, anecdotally some senior and sometimes specialist clinicians seem to reinforce student doubt, with comments suggesting that students are highly unlikely to encounter a range of rare conditions in general practice; this despite referrals to specialists often being from general practitioners. The perceived lack of relevance of rare conditions to future careers, undermines motivation to learn about anything other than the common.

Nonetheless, cursory examination of any pathology textbook, reveals a plethora of diseases that clinicians may encounter during their careers. A large proportion of conditions described in standard texts, have prevalence rates defining them as being rare; that is a prevalence less than or equal to 1 /1630 (less than or equal to 200,000 cases in the USA); 1/2000, or 1/2500 population, according to the USA, European Union and Japanese definitions respectively [11–13]. While rare diseases may be uncommon, they are many in number, so that from 3.5 to 5.9% of the population is thought to suffer a rare disease at any point in time [12].

A cohort of patients cared for by any given medical practitioner may be described as a ‘panel’. While the size of the panel varies depending on circumstance, it is reasonable to assume that many medical practitioners, serve panels of approximately 2000 patients [14]. From this, a general medical practitioner can expect to encounter roughly one case per year, of a spontaneously arising condition with a prevalence of 1/2000, the threshold for the European Union definition of a rare disease. Should a clinician practice for 30 years, then that single practitioner could expect to encounter one case of a disease during their career, with a prevalence of 1/60,000. Considering a cohort of 200 medical students, all graduating into 30 years of clinical practice, it would be expected that 12 graduates from that group, would encounter one patient each, suffering a condition with a prevalence of 1/1,000,000. Recalling that there are numerous diseases with very low prevalence, it becomes clear that the career numerical probability of encountering a patient with a rare disease is remarkably high. Because the authors serve a dental school, our particular interest lies in dental graduates. As such, we note that the same arguments apply for dental as do for medical graduates, while it is reasonable to estimate that dental panel size is around 1500 [15].

The historical success of the gambling industry testifies to poor general ability to accurately estimate numerical probabilities [16]. We argue that when students seek to balance the effort of studying rare diseases, against the need to acquire other clinically important knowledge, there is an underestimation of the relevant probabilities, and unhelpful skewing of learning away from otherwise important content. We further argue that carrying this habit post-graduation into clinical practice, may blind clinicians to noticing clinical manifestations of uncommon disorders.

A posture that resists notice of rare diseases seems reinforced by the academic community itself. When confronted with conflicting pressures on teaching time, seemingly superfluous content is readily weeded out. Under-estimation of the numerical significance of low-prevalence disease progressively narrows the scope of pathology courses. In a survey of physicians in Spain, less than a third of respondents reported they had received training in rare diseases during undergraduate or postgraduate studies [2]. One widely accepted approach to selecting which conditions should be studied by dental students, applies numerical scores for ‘commonness’, ‘significance’ and ‘uniqueness’, to select those conditions students are expected to become familiar with [17]. While this seems reasonable, it may also give both the students and faculty, false confidence in the scope of training offered, relative to ultimate clinical need.

It is impossible to know in advance, which of the many rare diseases, the patients of any given graduating clinician will suffer. However, it is entirely possible to use disease prevalence, to calculate the probability of clinicians encountering rare conditions [12].

This study aimed to develop and trial an educational intervention to raise student perception of the importance of learning about rare diseases. Our intervention was based on our exploration of the career exposure to conditions ranging from the very common to extremely rare, by computer simulation of clinical careers. We developed a class designed for first year dental students, which is structured around this computer simulation. We examined the effect of this on student perception of the importance of learning about rare diseases, by in-class on-line surveys conducted before and after the computer simulation.

## Methods

### Relating numbered theoretical conditions to reducing prevalence

**Fig. 1:**
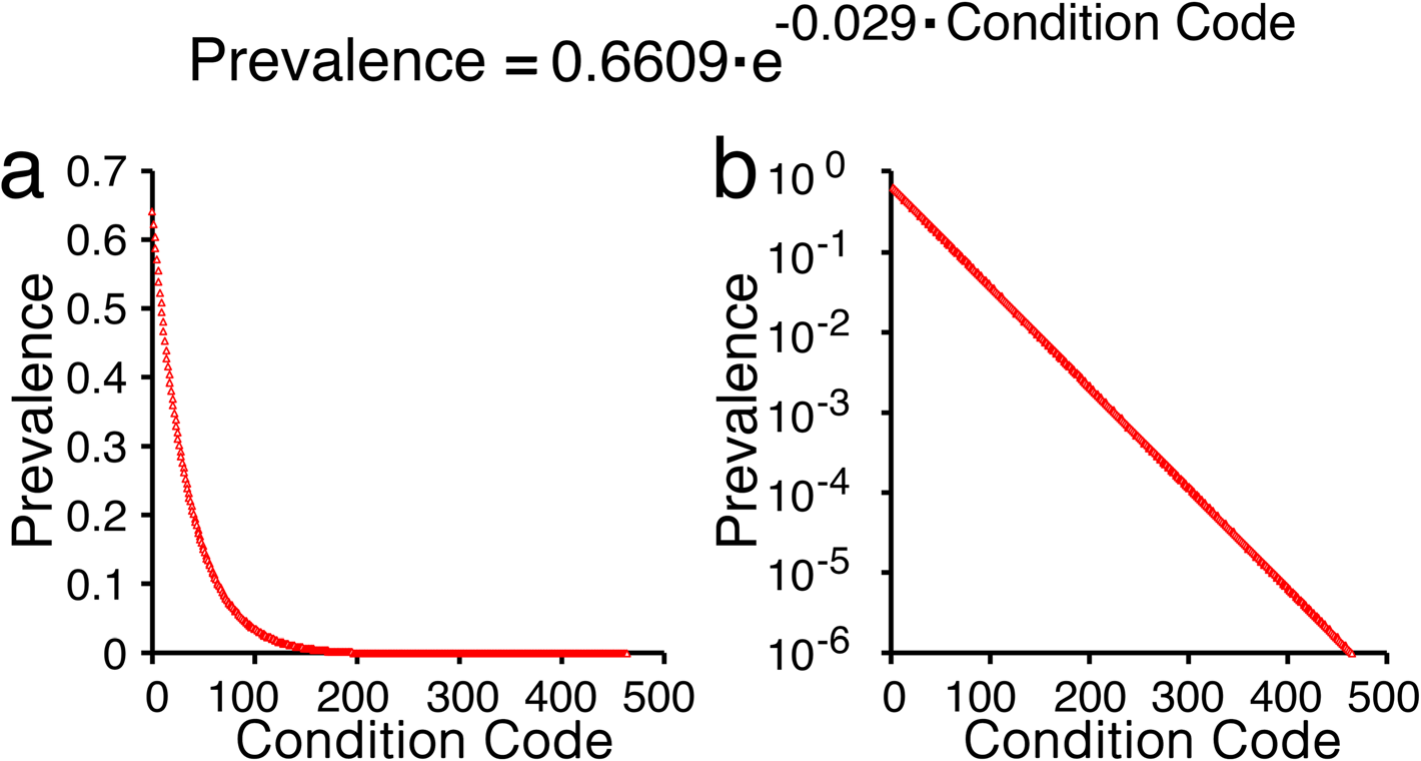
Graphs of the relationship between the CCN and calculated prevalence. The decaying exponential function shown was used to calculate prevalence values for theoretical conditions, each specified by a unique condition code numbered 1 to 462. Linear **(a)** and log **(b)** scales are shown. Condition code = 1 gave highest prevalence of 0.641; while condition code = 200 gave prevalence of 2 × 10^− 3^, which is a threshold below which conditions may be considered rare; condition code = 303 gave prevalence of 10^− 4^; condition code = 382 gave prevalence of 10^− 5^; and condition code = 462 gave prevalence of 10^− 6^

A theoretical ‘disease list’ was constructed of 462 discrete conditions, each identified by a Condition Code Number (CCN). A unique prevalence was assigned for each CCN as per the decaying exponential function shown in Fig. 1. Prevalence ranged from 1 per million, up to 64.1% of the population.

Each CCN was also randomly designated as representing either a spontaneously acquired condition (389 conditions) or a condition that is congenital and persistent (73 conditions), for example, haemophilia or amelogenesis imperfecta. The significance of this distinction is that congenital/persistent disorders must be handled in a numerically different way, compared with spontaneous diseases. This is because congenital/persistent conditions are carried life-long by patients, and can only be first encountered in new patients, either at the time the clinician starts work in a new practice or when a patient comes to a practice in replacement of other patients lost by attrition.

### Computer simulation of career experience encountering conditions with varying prevalence

**Fig. 2:**
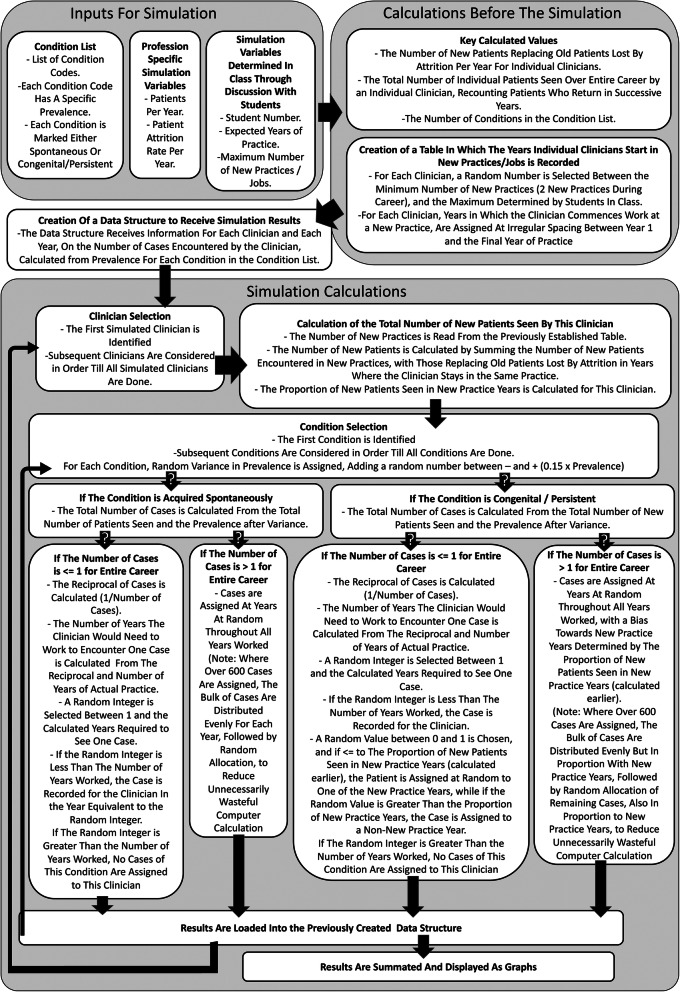
Algorithmic design for career experience of conditions with varying prevalence for a graduating cohort. Inputs for the simulation are defined as either: from a condition list; specific to the health profession involved; or offered by students in-class. Important universal values for the simulation are calculated from inputs, and a data structure created to receive results. The simulation involves two separate loops, the outer loop cycles from the first to the last simulated clinician. The inner loop cycles for each clinician through the list of conditions according to CCN. Important ‘if statements’ leading to different handling of prevalence values are indicated with ‘?’ in arrows. These test: whether the specific CCN is classified as being acquired either ‘spontaneously’, or as a ‘congenital/persistent’ condition; and if the expected number of cases for the particular CCN is less than 1. Once all clinicians and conditions have been considered, data are summated and displayed graphically

Simulation script was developed in MATLAB (MATLAB by MathWorks Inc) according to the algorithmic approach detailed in Fig. 2. MATLAB simulation scirpt is provided in Supporting Information. The simulation was of the career of each clinician in turn, considering each condition specified by CCN also in turn. For each CCN, cases representing individual patients were assigned at random and according to probability calculated from prevalence, to individual years of practice. To mimic variation in prevalence due to local geographic and demographic factors, prevalence for each CCN and clinician was modified, by addition or subtraction of a random number between 0 and 15% of the respective prevalence value.

### Class plan with in-class surveys before and after in-class computer simulation

**Fig. 3:**
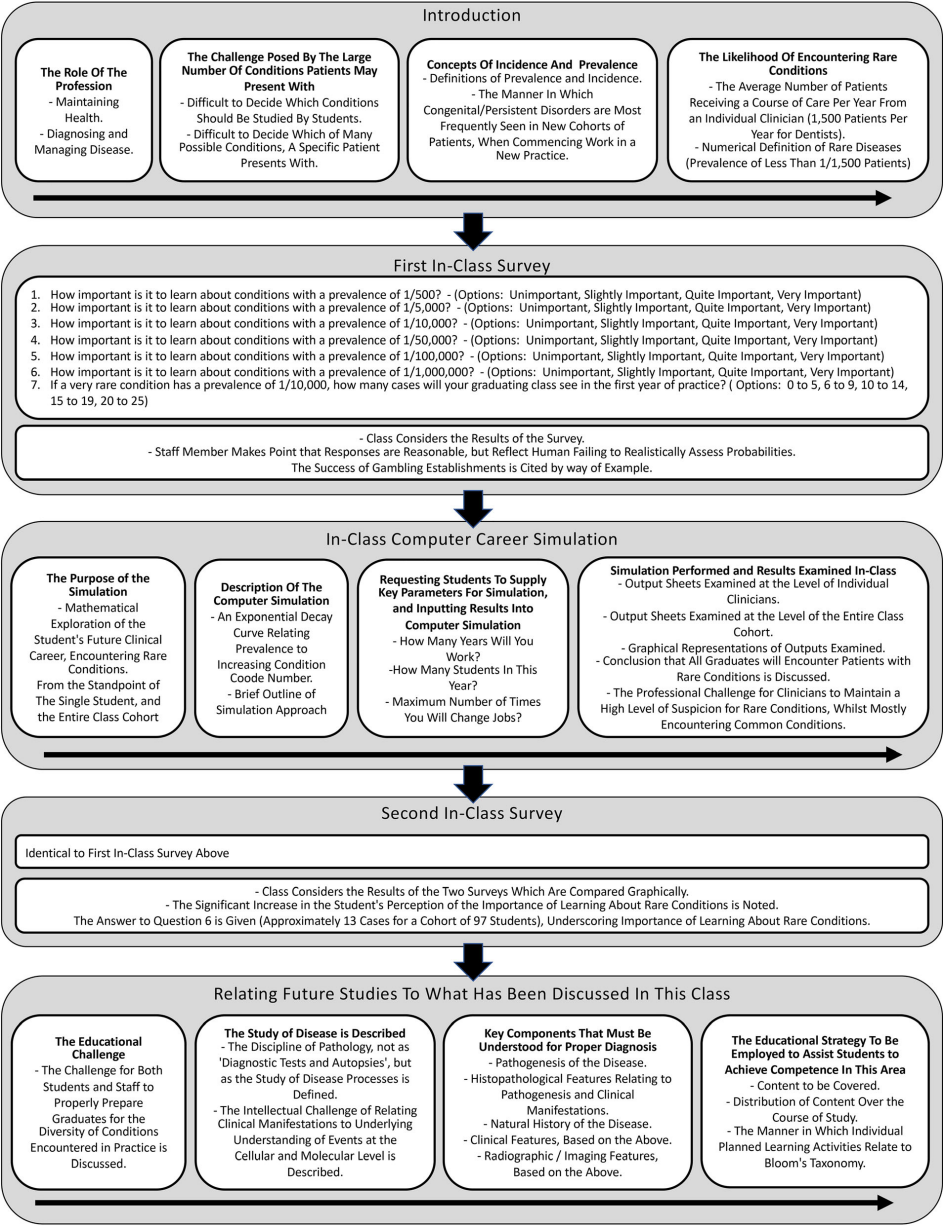
Flow chart illustrating the class plan. An introduction was followed by an in-class on-line student survey determining student perception of the importance of learning about rare conditions. Results of the survey were briefly examined. An in-class computer simulation was then performed. The simulation yielded the number of patients that the current class can expect to encounter throughout the entirety of their clinical careers, who suffer discrete conditions ranging from high to negligibly low prevalence. Results were examined at the level of individual clinicians, as well as for the entire class cohort considered together. The in-class survey was then repeated, noting any appreciable change in perception of the importance of learning about rare conditions. The educational strategy employed to assist students in their future learning, was then discussed

A 1.5 h class titled ‘A career full of surprise’ was designed and delivered according to the flow-chart in Fig. 3. This was a new class that had not previously been delivered, and was for first year dental students in their first week of dental studies. The class was timed to precede within weeks, first classes on dental developmental defects, many of which are rare diseases. We felt that this order and close proximity of the two learning experiences would mutually reinforce the learning impact of both. Furthermore, the curriculum we teach delivers most fundamental Oral Pathology in the first 2 years of the course, and we felt it was prudent to try and raise student interest in rare diseases at an early stage, rather than for students to feel they had started paying attention to rare diseases ‘too late’ towards the end of the curriculum. A 1.5 h class time was selected, on the grounds that it provided sufficient time to cover the new material, and that a more extended session ran the risk of losing student attention.

Learning objectives were to: be able to define the terms ‘incidence’ and ‘prevalence’, and to explain the difference between these two; to be able to define the term ‘rare disease’; to be able to explain how patients with rare congenital and persistent diseases, are most likely to be encountered in a panel of patients during the first year of practice in a new location; to be able to correctly estimate the likelihood of encountering patients with rare diseases throughout the length of a clinical career. While concepts of incidence and prevalence had been introduced to previous student cohorts in other classes, this was the first occasion where the likelihood of encountering patients with rare diseases was taught.

An introduction leading to the question of the importance or otherwise of learning about uncommon diseases was followed by a short in-class on-line survey on the importance of learning about rare conditions, similar to the use of survey tools by others [18–21].

Results of the survey were then graphed, examined and discussed in class. It was important for the staff member to state low priority to learning about rare conditions reflected natural human deficiency estimating probabilities, exploiting the power of discordant observations to impress memory [22]. The computer simulation was then described. To help students project themselves into the scenario, students were asked to provide three key variables for the simulation: student cohort number (students reported 95 in cohort); number of years they plan to work (consensus amongst students was 40 years); and the maximum number of times they anticipate changing jobs (consensus amongst students was 10 new jobs). The simulation was then run. Results were then examined in-class: firstly, considering tabulated single year and entire career results for individual clinicians (as in Supporting Information); then considering tabulated results for the cohort as a whole (as in Supporting Information), and finally examining scattergrams. The in-class on-line survey was then repeated, and results graphed for comparison with results of the first survey. Peer pressure impacts learning [23–25], and visual demonstration that most students had increased perception of the importance of learning about rare conditions, was exploited to encourage students still unimpressed, to raise their interest to the level of their colleagues.

The first year cohort contained 94 students. Although no roll was taken, there were 89 respondents to the first survey, and 77 respondents to the second survey.

Active enlistment into a shared endeavour increases commitment [24]. To that end, there was then discussion of the educational challenge for both students and academics, in understanding and addressing the diversity of disease, from the perspective of learner and teacher both. Also discussed, was the educational approach that would be taken, to assist students in their learning.

## Results

### The computer career simulation demonstrated that all members of a graduating student cohort, could expect to frequently encounter patients with rare diseases

**Fig. 4:**
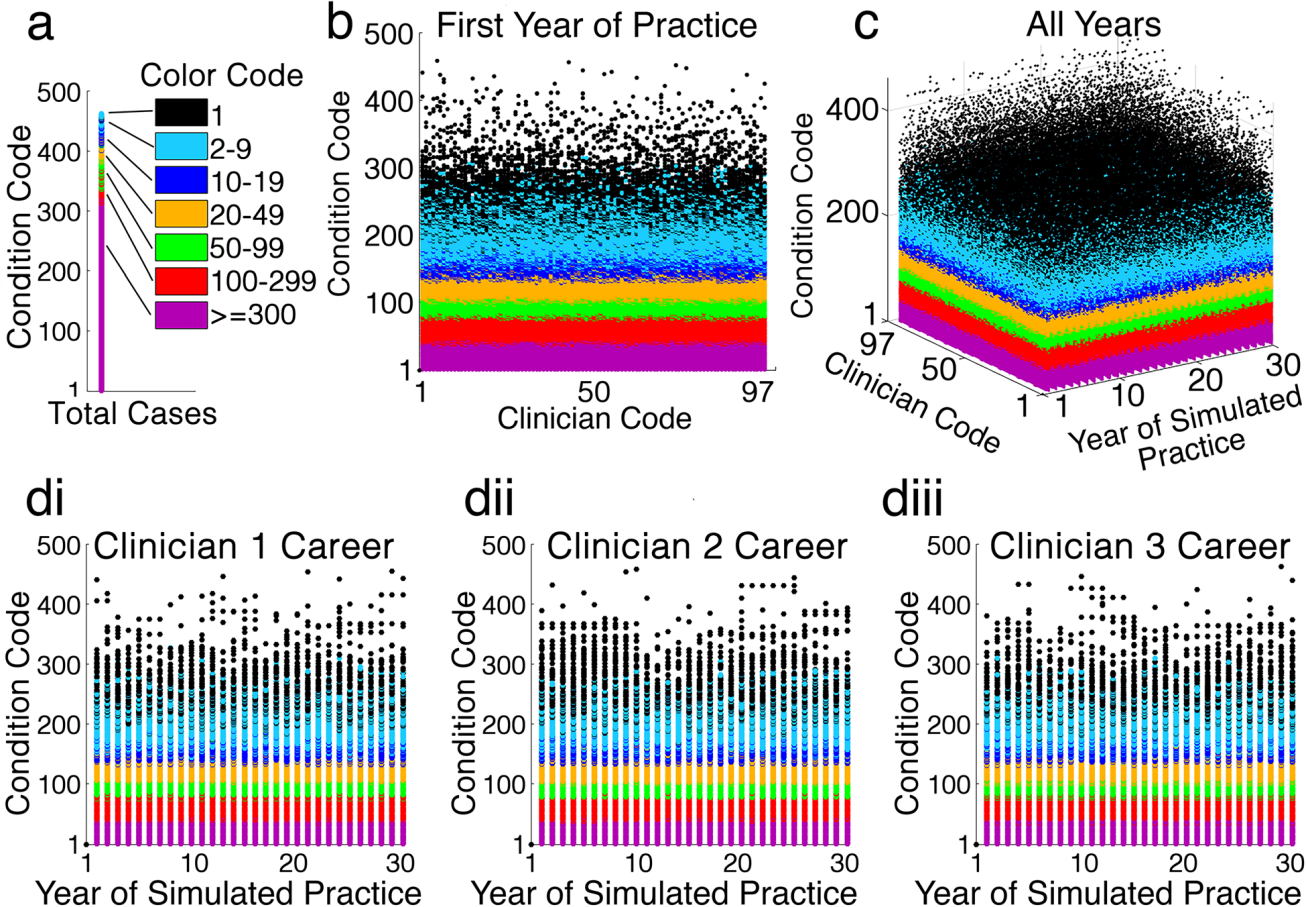
Scattergrams of a typical career simulation for a cohort of graduating students. Ninety-seven students were simulated, practicing for 30 years, and changing practice from 2 to 10 times. Each dot shown indicates the number of cases seen by colour as per the given colour code (1 case is black, through to > = 300 cases as magenta). **a** The total number of cases of each condition across all years for the entire simulated clinician cohort is shown. Relating condition code to prevalence as per Fig. 1, many simulated patients with rare conditions were encountered by the entire simulated cohort in their combined careers. **b** Results for the entire simulated cohort in their first year of practice are shown. It is clear that the simulated cohort encountered a wide range of rare conditions even in their first year of practice. **c** Results for all simulated clinicians, all years of practice, and all conditions are shown. Even conditions with extremely low prevalence were encountered by the simulated graduated cohort throughout their careers. **d** Results for the entirety of the careers of the first three clinicians are shown (di, dii, diii). Condition codes with low prevalence appearing in multiple consecutive years, represent simulated conditions that are congenital/persistent. These are only lost when the simulated clinician changes practice. Throughout the entirety of the simulated careers shown, each simulated clinician encountered numerous rare conditions

Fig. 4 summarizes results of a typical simulation for the 30-year careers of 97 simulated dental clinicians (tabulated detailed numerical output in Supporting Information) Comparison of the prevalence for each condition in Fig. 1, with the location of marks in scattergrams in Fig. 4, clearly demonstrates that each simulated clinician encountered many patients with rare conditions during their careers. It was further evident that the cohort as a whole encountered almost every condition modelled, at least several times.

### The computer career simulation increased student perception of the importance of learning about rare diseases

**Fig. 5:**
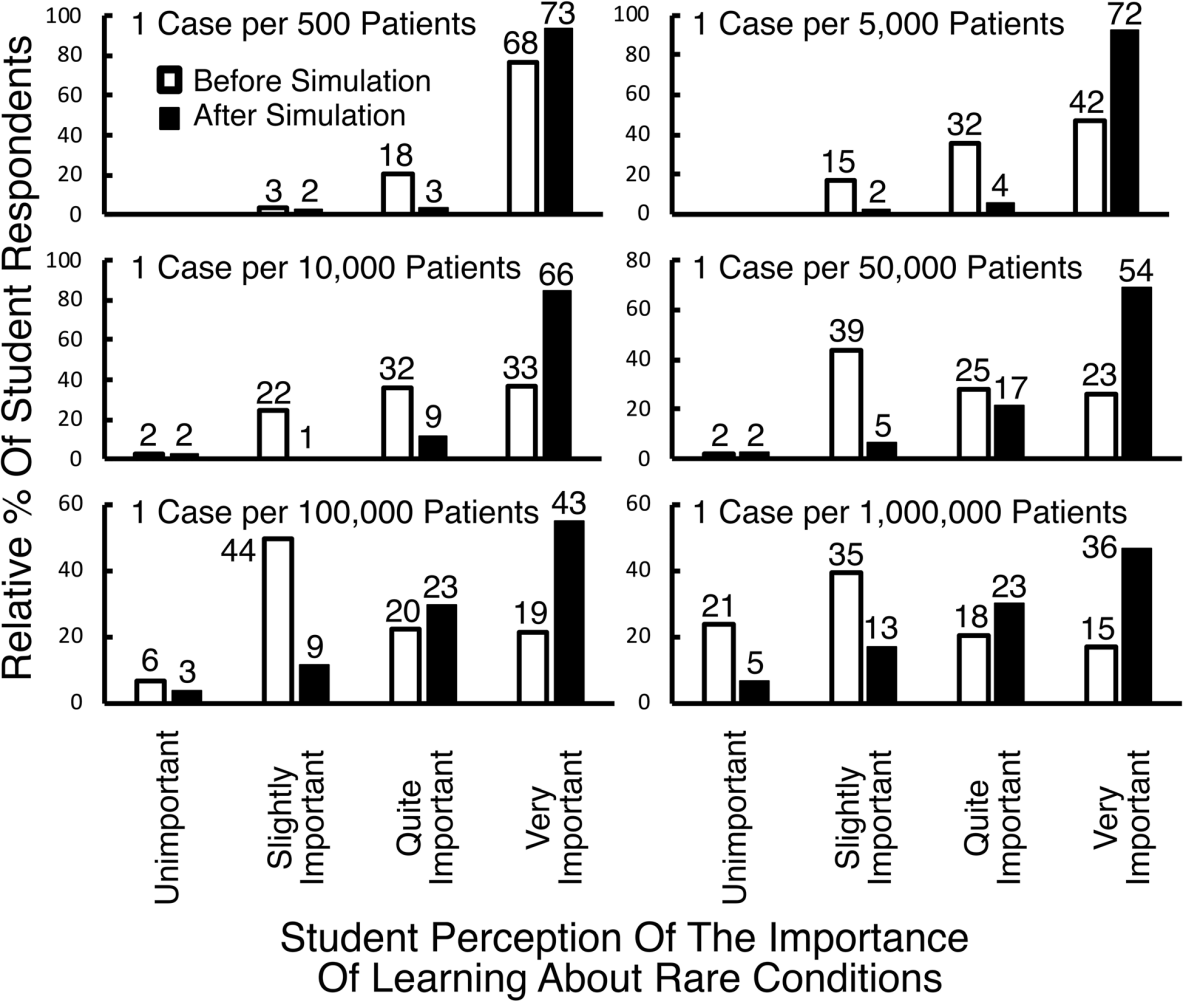
Results of in-class on-line surveys on perception of the importance of learning about rare conditions. Students were asked about their perception of the importance of learning about conditions with differing prevalence, both before (open bars) and after (filled black bars) the in-class computer simulation. Both relative percentages (bars) and numbers of respondents are provided (over bars). Students were asked their opinion on how important it is to learn about conditions with prevalence ranging from 1/500 patients, to 1/million patients. There were 89 respondents to the first survey, and 77 respondents to the second survey. Statistical significance was evaluated by the Chi-Squared test, grouping results for ‘unimportant’ with ‘slightly important’, as well as results for ‘quite important’ and ‘very important’. While there was no significant change in student perception of the importance of learning about conditions with a prevalence of 1/500 patients, a clear increase in perception of the importance of learning about rare conditions was seen for conditions with progressively lower prevalence (*p* < 0.005 for 1/5000 patients; *p* < 0.0001 for 1/ 10,000 to 1/1,000,000 patients)

Comparison of survey results before and after computer simulation (Fig. 5), suggest a statistically significant increase in the perceived importance of learning about rare diseases by students, after the computer simulation (*p* < 0.005, Chi-squared Test). Table 1 shows a similar change in the response to a question estimating the number of cases the cohort would encounter in their first year of practice, of a condition occurring with a prevalence of 1/10,000 (*p* < 0.0001, Chi-squared Test).

**Table 1:** Student responses before and after the computer simulation, to the question: *‘If a very rare condition has a prevalence of 1/10,000, how many cases will your graduating class see in the first year of practice?’*

Number of Expected Cases	Number and (Relative Percentage) of Respondents	
	Before Simulation	After Simulation
**0 to 5**	35 (39.3%)	7 (9.1%)
**6 to 9**	16 (18.0%)	6 (7.8%)
**10 to 14**	20 (22.5%)	17 (22.1%)
**15 to 19**	15 (16.9%)	25 (32.5%)
**20 to 25**	3 (3.4%)	22 (28.6%)
**Total**	89 (100%)	77 (100%)

The calculated correct answer from the simulation was ‘13’, and there was no significant change in the relative percentage of students selecting the answer in the correct range. Of more interest, however, was that after the computer simulation, students displayed clear tendency to over-, rather than under-estimate, the number of patients the cohort would encounter in the first year of practice (*p* < 0.0001, Chi squared test grouping results for ‘0 to 5’ and ‘6 to 9’, and comparing with grouped results for ‘10 to 14’, ‘15 to 19’, and ‘20 to 25’)

## Discussion

We here describe our exploration of the career exposure to conditions ranging from the very common to extremely rare, by computer simulation of clinical careers, and demonstrate the inevitability of practising clinicians frequently encountering patients who have rare diseases. We also describe a class designed for first year dental students, which is structured around this computer simulation. The class aims to leverage student engagement via the inclusion of simulation inputs given by students in class and real-time in-class surveys, to generate student discussion and help students project themselves into the simulation. We further report the effect of this, increasing student perception of the importance of learning about rare diseases, as assessed by in-class on-line surveys.

Others have conducted surveys to try and identify priorities and approaches for teaching physicians about rare diseases [4, 26], as well as students [5–9]. One suggestion is for there to be education directed to identifying ‘red flags’ [4]. Engagement with television shows in which rare diseases feature, has also been explored [9]. The approach outlined by us aims at increase awareness of the probability of encountering rare conditions, and appears to be novel.

Apart from this new computer simulation based class, our practice has been to weave learning about both common and rare diseases, into thematic blocks focused on logical groupings of diseases. We structure learning through a teaching tool we have developed and term ‘Clinico-Pathological Enquiry Based Learning Sessions’ (CPEBLS). These are blended learning experiences, with each CPEBLS devoted to a distinct group of conditions, such as ‘dermatological conditions affecting the oral mucosa’, or ‘bony dysplasias’. Each CPEBLS exercise comprises discrete stages: i) an introductory lecture that provides an overview of relevant material; ii) an exercise in which students collect information from texts on discrete diseases under structured headings such as ‘pathogenesis’, ‘histopathological features’, ‘clinical features’, and ‘further investigations’; iii) students then apply the gathered information to answer questions that require interpretation of clinical images, virtual microscopy slides, radiographs and or clinical scenarios, as well as hypothesis generation; iv) answers given to questions in stage (iii) are then discussed with a staff member in a review class; v) and each CPEBLS exercise is concluded with a formative student self-assessment via an on-line examination tool. Our emphasis throughout is on developing skill and confidence in explaining clinical and other features of all diseases, from first principles. Rare diseases that we include in CPEBLS are those that either provide an excellent illustration of some important principle, or alternatively have special clinical significance. Although it is impossible for us to ‘cover’ all rare diseases in this way, we believe this blended strategy focused on explanatory principles, provides our graduates with a sound basis for identification of both common and rare diseases, based on the rational application of first principles. With regard to the subject of the current paper, our equating in CPEBLS, the importance of rare conditions with those that are common, reinforces the importance of rare diseases. Outcomes from examinations in junior years are favourable for Oral Pathology, and we see retention and maturation of Oral Pathology knowledge throughout the senior years.

Although our in-class survey results suggested that the computer simulation comprised an effective educational intervention that increased perceived importance of learning about rare conditions, this may not be carried forward throughout further training, and additional studies are required to monitor this. As such, these results must be accepted as preliminary, and no statement can be made on possible persistence of the observed effect. It would be valuable to apply this survey again to this student cohort at the conclusion of senior studies, while it would also be interesting to similarly compare results for the survey of senior students, with those of the first year cohort.

The computer simulation confirmed the comparatively frequent encounter of rare conditions by clinicians, however, there were some significant limitations. For example, it may have been more illustrative for the simulation to have drawn from a list of specific diseases with known prevalence rates, rather than from the decaying exponential function used. Unfortunately, unambiguous prevalence values are currently unavailable for surprisingly many conditions, and we are currently seeking to overcome this.

A further limitation of the simulation approach, is division of conditions into only two types, being those acquired spontaneously, and others that are congenital/persistent disorders. This may not properly model conditions that arise spontaneously, but which then persist life-long. Nonetheless, application of prevalence values in the way used in the simulation, does seem to make reasonable accommodation for this.

Apart from variation in the number of times clinicians commence work in new practices, all clinicians are modelled as having identical career pathways. The careers of specialist clinicians, who may care for differing numbers of patients per year, and who also encounter mostly new patients in each year, are not modelled. While this might be an interesting future refinement, we do not think it would add significantly to the illustrative value of the simulation. We similarly feel that allowing for differing career length, or interruptions to careers amongst simulated clinicians, would have little impact on utility of the simulation.

Since several of us are based in a dental school, our specific interest lies in dental students. However, we see that our approach and computer simulation is equally applicable for students across other health disciplines, including medicine, the allied health professions, and veterinary science.

Currently, delivery of the class requires not only familiarity with MATLAB coding, but also in-class use of several different computer programs: PowerPoint (Microsoft) for slide presentation; MATLAB for computer simulation; poll everywhere (polleverywhere.com) and a web-browser for the on-line survey; and Excel (Microsoft) for graphical display of survey results. Development of a program that combines these elements into a single user-friendly format would be helpful. Such a format should ideally include: the opportunity to load discrete disease lists specific for the profession and student cohort involved; capacity to select input variables for patient number and attrition rate specific for each professional group, and ability to load student email lists for automated circulation to students of the simulation and survey results.

While computer simulations are widely used in medical education [27–30], we are unaware of computer simulation having been previously used to model careers in the way shown in this report. We imagine our approach will be of interest to others seeking to motivate students towards increased commitment to study both rare and moderately uncommon diseases.

We also hope the results of our computer simulation, will lead some of our academic colleagues to reconsider what seems to be progressive narrowing of academic content, in response to increasing pressure on curriculum time. The helpfully instructive aphorism ‘when you hear hoofbeats behind you, don’t expect to see a zebra’ [31], was never meant to suggest consideration of horses alone.

## Conclusion

We conclude that in-class computer simulation of career occurrence of patients suffering rare diseases, provides an effective strategy to re-align student under-estimation of rare conditions with community and educational needs.

## Supplementary Information

**Additional file 1.**

**Additional file 2.**

## Data Availability

Data is made available in the manuscript and supplementary information. Similarly, full MATLAB code required to perform the computer simulation is provided in supplementary information.

## Abbreviation

CCN: Condition code number
