# Supplementary material for: Career-computer simulation increases perceived importance of learning about rare diseases
Source: BMC Med Educ. 2021 May 17;21:279. doi: 10.1186/s12909-021-02688-7 (PMC8127215; doi:10.1186/s12909-021-02688-7)
Supplement: Supplementary file 1 — Additional file 1. [file 12909_2021_2688_MOESM1_ESM.pdf]

Supplementary Material for:

Career-computer simulation increases perceived importance of learning about rare diseases

Babak Sarrafpour[1], Shwetha Hegde[2], Eduardo Delamare[2], Ruth Weeks[3], Rebecca A. Denham[3], Alix Thoeming[3], Hans Zoellner[1,4]

[1] The Cellular and Molecular Pathology Research Unit, Oral Pathology and Oral Medicine, Discipline of Oral Surgery, Medicine and Diagnostics, The School of Dentistry, The Faculty of Medicine and Health, The University of Sydney, Westmead Centre for Oral Health, Westmead Hospital, Westmead, NSW 2145, Australia

[2] Radiology, Discipline of Oral Surgery, Medicine and Diagnostics, The School of Dentistry, The Faculty of Medicine and Health, The University of Sydney,
Sydney Dental Hospital, Surry Hills, NSW 2010, Australia

[3] Educational Innovation Team, The Fisher Library Stack, The University of Sydney, Camperdown, NSW 2006, Australia

[4] Biomedical Engineering, Mechanical Engineering Building, Faculty of Engineering, Darlington Campus, the University of Sydney, NSW 2006, Australia

MATLAB Script

```
% SCRIPT FOR ESTIMATION OF THE NUMBER OF PATIENTS PRESENTING WITH
% CONDITIONS OF KNOWN PREVALENCE, TO INDIVIDUAL CLINICIANS IN A COHORT OF
% PRACTITIONERS OVER A DEFINED PERIOD OF TIME
% (Script37AllGrph.m - 12 July, 2019)

%-----------------------------------------------------------------
% INPUT DATA VARIABLES
% PtsYr = Number of patients treated per year by one clinician
PtsYr = 1500;

% a = Percentage attrition and replacement rate of patients per year
Atrit= 17;

% y = Years of practice
year = 44;

% StNo = Student number
StNo = 95;

% ChJMax = Maximum Number of new full time practices any single clinician
%     can start work at = NOTE:ChJMax <= 14
ChJMax = 15;

% dNo = Number of diseases (conditions) considered, determined from the
%     disease list

%----------------------------------------------------------------
% DATA FILE THAT MUST BE LOADED
% DisLst = List of diseases imported as a numeric, in which:
%     Column 1 = Disease code number
%     Column 2 = Available to calculate variable range if desired
%     Column 3 = Prevalence value (Range between 0 and 1)
%     Column 4 = Congenital condition indicated by '1'
%                Acquired condition indicated by '0'
%     NOTE: Should be imported as a numeric matrix

DisList = Diseases;


%---------------------------------------------------------------
% ACTIVATING OR INACTIVATING 'TWISTER' FOR REPRODUCIBLE RANDOM NUMBERS
% By inserting or deleting '%', before the following active line,
%     random number generation can be made either reproducible
%     (% deleted), or not ('%' inserted)

%rng(0,'twister');

%--------------------------------------------------------------
% CALCULATION OF IMPORTANT VALUES

% Number of new patients replacing those who leave the practice per year
NewPtsYr = PtsYr*Atrit/100;

% Number of diseases (dNo)
MSz= size(DisList);
dNo=MSz(1,1);
```

```
clear MSz

% Variance Range (VarRng) for each disease (Maximum value that can be
%     added or subtracted from 1 to give the number by which the
%     prevalence is multiplied - generating reasonable variability
%     through application of an exponential function with minimum
%     variance at 0.15, and maximum rising towards 0.8 with reducing
%     prevalence. This is inactivated by insertion of '%'.
%     Instead, a flat '0.15' variance is otherwise applied
% for i=1:dNo
%    P = DisList(i,3);
%    VarMlt = 500000000^-((P + 0.05)) + 0.15;
%    DisList(i,2) = VarMlt;
% end

%----------------------------------------------------------------
% DEFINING YEARS THAT CLINICIANS START IN NEW PRACTICES
% Creating a matrix 'Clin' to receive results
Clin = zeros(StNo,15);
Clin(:,1)=1;

% Loading results into Clin.  All clinicians start in Year 1, and have
%     random assignment of new jobs after that up to 'year'

for st = 1:StNo
    jbs = randi([2,ChJMax]);
    if jbs < year
        div = year/jbs;
        div = round(div);
        count = 0;
        for i = 1:jbs
            a = div*count;
            b = div*(count+1);
            yr = randi([a,b]);
            Clin(st,i+1) = yr;
            if yr >= year
                Clin(st,i+1 )= 0;
            end
            count = count + 1;
        end
    end
end

% Unhelpful '1' and '0' values are placed in Column 2 by the above
%      and these are now cleaned out
for i = 1:StNo
    if Clin(i,2) == 0
        a = Clin(i,3);
        b = round(a/2);
        Clin(i,2) = b;
    end

    if Clin(i,2) == 1
        a = Clin(i,3);
        b = round(a/2);
        Clin(i,2) = b;
    end
end

clear st jbs div count i b yr a c cnt
```

```
%---------------------------------------------------------------

% PREPARING RESULTS MATRIX
% A 3D data matrix (Mat) is prepared to receive and summarise results

% EACH 2D 'SHEET' represents a single year of  clinical practice,
%      indexed from Year 1 to year y.

% For each year (2D sheet), Rows = Individual Diseases (= dNo) and
%      Columns = Individual Clincians (= StNo+1).

% Clinicians (Columns) are indexed 1 to StNo from left to right

% Creating matrix for rresults (Mat)
Mat(1:dNo,1:StNo,1:year)=0;

%---------------------------------------------------------------

% DETERMINING THE NUMBER OF PATIENTS SEEN BY INDIVIDUAL CLINICIANS
% FOR EACH DISEASE ACROSS ALL YEARS AND DISTRIBUTING TO YEARS

% Each clinician is considered in order.  Firstly, the years that
%    individual clinicians change practice are identified, as this
%    has bearing on the number of new patients encountered.

% The total number of new patients seen by the clinician throughout their
%    career is calculated, taking into account the number of times
%    practices are changed.

% Acquired conditions arise sporadically across the population
%    in proportion to the prevalence rate, so that it is fair to assign
%    acuired conditions in proportion to the number of patients seen
%    per year.

% Congenital conditions, however, are present throughout the life of
%    the patient.  For this reason, only new patients are assumed to
%    present with congenital disorders. Such new patients are encountered
%    in greatest number in the first year when starting at a new
%    practice.  In remaining subsequent years within the practice,
%    congenital disorders can only be encountered in new patients
%    joining the practice in replacement of those patients who leave.
%    The below script accommodates this effect, distributing congenital
%    disorders in proportion to new patients seen, either in the first
%    year of a new practice, or replqcing patients lost through attrition.

% For acquired conditions, the number of patients seen (on a yearly basis)
%    throughout the career of the clinician is calculated.

% For congenital conditions, the number of new patients seen throughout
%    the career of the clinician is calculated.

% Two approaches are taken in generating reasonable vasriance.
%     In one, an exponential function is applied that generates increasing
%     maximum variance with reducing prevalence. There is andom selection
%     of possible final prevalence values between a maximum and minimum
%     that expands further from the 'base prevalence' as base prevalence
%     reduces. This generates 'strings' of high values that appear odd
%     in graphical representation.
```

```
%     In the other, a flat maximum and miniumum variance of 0.15 of the
%     base prevalence is established, and random selection is made
%     between these two values. This generates more convincingly regular
%     graphical representations.

% The relevant number of patients (dependent if acquired or congenital
%    disease is considered) is multiplied by the prevalence rate to
%    estimate the number of expected patients with the condition.
%    If the number of patients expected is less than 1, the recipicol
%    of the number of patients is determined, and used to multiply
%    the relevant patient number to establish the number of patients
%    who would have to be seen in order to encounter one case.

% For conditions with low prevalence (<=1 Cases), a recipricol value
%    is calculated from the prevalence after adjustment for variance
%    as described above.
%    For acquired conditions, a random integer is selected between 1 and
%    the product of the reciprocol of the number of cases and the number
%    number of practice years. If this is less than the number of
%    practice years, a case is assigned to the year identical to the
%    random integer.
%    For congenital conditions, the reciprocol is also used as above
%    to determine if a case was assigned to the clinician, but using
%    the correct number of total patients relevant to congenital
%    conditions. It is then decided if the case is to be assigned
%    to a 'new practice year', or a year between new jobs. This is
%    done by comparing the ratio of new practice years relative to
%    total years practiced, with a random number between 0 and 1.  If
%    the random number is less than the 'new practice year ratio', the
%    case is assigned to a new practice year, randomly selected from
%    amongst the years the individual starts a new job.  If the random
%    number is above the calculated ratio, the patient is assigned to
%    a random year other than when starting at a new practice.
%    Patients with congenital disorders are assumed to continue returning
%    to the same practice throughout the tenure of the clinician, so
%    each subsequent year in the practice is assigned the same patient
%    again.

%    For conditions with Cases > 1 and <= 600, a different approach
%    is used.
%    For acquired conditions, individual cases are assigned randomly to
%    years of practice.
%    For congenital conditions, cases are assigned in an identical way
%    to that outlined above for low prevalence (Cases <= 1), with the
%    difference that more than one case is assigned.

%    For conditions with > 600 cases, it is assumed that there is
%    negligible significance if the condition is acquired or congenital,
%    so both are handled in the same way. To make simulations reasonably
%    speedy, the bulk of cases are assigned in equivalent number to
%    all years equally, and then remaining cases are randomly assigned
%    to years.


% Total number of patients seen on a per clinician per year basis
AllPtPrSt = PtsYr*year;

% COMMENCEMENT OF A LOOP ASSIGNING CASES TO CLINICIANS ACROSS YEARS
```

```
for st=1:StNo
    %----------------------------------------
    % DETERMING WHICH YEARS THE CLINICIAN STARTS AT A NEW PRACTICE
    % Counting the number of jobs from Clin
    cnt = 0;
    for i = 1:ChJMax
        if Clin(st,i) > 0
            cnt = cnt + 1;
        end
    end

    % Constructing and filling matrix of years starting practice 'Jobs'
    Jobs = zeros(1,cnt+1);
    for i = 1:cnt
        Jobs(1,i) = Clin(st,i);
    end
    Jobs (1,cnt+1) = year;

    % The number of new patients encountered in first year working at
    %     new practices (NewPracPts)
    NewPracPts = cnt * PtsYr;

    % Total number of new patients encountered during career
    %     - NOTE: NewPtsYr = Patients replacing attrition
    AtRepPts = (year - cnt) * NewPtsYr;

    % Total new patients throughout career
    TotPts = NewPracPts + AtRepPts;

    % Proportion of cases of congenital-persistent diseases to be
    %       distributed to New Practice Years (NPY)
    NPY = NewPracPts/TotPts;

    for di=(1:dNo)
        %-------------------------

        % VARIATION OF PREVALENCE
        % The exponential function can be applied as per:
        %    vmax = 1 + DisList(di,2);
        %    vmin = 1 - DisList(di,2);
        %    PrevV = vmin + (rand()*(vmax-vmin));
        %    Prev = PrevV * DisList(di,3);

        % The flat +/- 0.15 variance can be inactivated by inserting
        %     '%' symbols in the below script
        min = 1.15;
        max = 0.85;
        var = min + rand()*(max - min);
        Prev = var * DisList(di,3);


        % FOR ACQUIRED CONDITIONS
        % Number of cases according to prevalence

        if DisList(di,4)==0
            Cases = Prev*AllPtPrSt;

            if Cases <= 1
                Recip= 1/Cases;
```

```
            d = year*Recip;
            e = round(d);
            c = randi([1,e]);
            if c <= year
                Mat(di,st,c)=(Mat(di,st,c))+1;
            end
        end


        if Cases >1 && Cases <= 600
            for i= (1:Cases)
                x=randi([1,year]);
                Mat(di,st,x)=(Mat(di,st,x))+1;
            end
        end

        if Cases > 600
            % Bulk allocation
            CpY = Cases/year;
            b = Cases - (2*CpY);
            g = b/year;
            g = round(g);
            rem = Cases - (g*year);
            Mat(di,st,:)=g;
            for i=1:rem
                y = randi([1,year]);
                Mat(di,st,y) = Mat(di,st,y)+1;
            end
        end

    end


    %--------------------------------------------
    % FOR CONGENITAL & OR PERSISTENT CONDITIONS
    % Number of cases according to prevalence
    if DisList(di,4)==1
        Cases = Prev*TotPts;

        if Cases <= 1
            Recip= 1/Cases;
            d = year*Recip;
            e = round(d);
            c = randi([1,e]);

            if c <= year
                r = rand(1);
                if NPY <= r
                    a = randi([1,cnt]);
                    ys = Jobs(1,a);
                    g = Jobs(1,a+1)-Jobs(1,a);
                end

                if NPY > r
                    bx = randi([1,cnt]);
                    f = Jobs(1,bx);
                    s = Jobs(1,bx+1);
                    ys = randi([f,s]);
                    g = s - ys;
```

```
                end
                Mat(di,st,ys) = Mat(di,st,ys)+1;

                for i = 1:g
                    Mat(di,st,ys+i) = Mat(di,st,ys+i)+1;
                end
            end
        end

        if Cases > 1 && Cases <= 600

            for e = 1:Cases
                r = rand(1);
                if NPY <= r
                    a = randi([1,cnt]);
                    ys = Jobs(1,a);
                    g = Jobs(1,a+1)-Jobs(1,a);
                end
                if NPY > r
                    bx = randi([1,cnt]);
                    f = Jobs(1,bx);
                    s = Jobs(1,bx+1);
                    ys = randi([f,s]);
                    g = s - ys;
                end
                Mat(di,st,ys) = Mat(di,st,ys)+1;
                for i = 1:g
                    Mat(di,st,ys+i) = Mat(di,st,ys+i)+1;
                end
            end
        end


        if Cases > 600
            % Bulk allocation
            CpY = Cases/year;
            b = Cases - (2*CpY);
            g = b/year;
            g = round(g);
            rem = Cases - (g*year);
            Mat(di,st,:)=g;

            for i=1:rem
                y = randi([1,year]);
                Mat(di,st,y) = Mat(di,st,y)+1;
            end

        end
    end
  end
end


%--------------------------------------------------------------
% SUMMATTING RESULTS

% A matrix (SingClin) records the total number of patients
%      (counted as patients / year) for each condition seen.
%      (Column number = Clinician, Row number = disease)
```

```
% A separate matrix (TotalAllClin) gives totals for each disease (Rows)
%      across all Clinicians in each year (Columns), while
%      the final Coliumn (at Years +1), gives total for all clinicians
%      and all years

% Two further separate matrices (ClinOne and ClinTwo) show results for
%      the first two clinicians - Mat(:,1,:) and Mat(:,2,:)

% NOTE: Values are in units of patients per year
%      From this patients with congenital conditions are
%      re-counted for every year they are present in the practice

%----------------
% Creating a matrix (TotAllClin) to receive results
TotAllClin = zeros(dNo,year+1);

% Summating individual years
t=0;
for  Page=(1:year)
    for Row=(1:dNo)
        for Col=(1:StNo)
            t= t+Mat(Row,Col,Page);
        end
        TotAllClin (Row,Page) = t;
        t=0;
    end
end

% Summating all years for all clinicians
t=0;
for Row=(1:dNo)
    for Col=(1:year)
        t= t+TotAllClin(Row,Col);

    end
    TotAllClin(Row,year+1) = t;
    t=0;
end

%---------------
% Creating Matrix (SigClin)
SingClin =zeros(dNo,StNo);

% Summating Years for SingClin
t=0;
for Row=(1:dNo)
    for Col=(1:StNo)
        for Page=(1:year)
            t= t+Mat(Row,Col,Page);
        end
        SingClin(Row,Col)=t;
        t=0;
    end
end

%---------------
% Creating Matrix (ClinOne)
ClinOne = zeros(dNo,year);
```

```
% Loading results from Mat(:,1,:)
for y = 1:year
    for d = 1:dNo
        ClinOne(d,y) = Mat(d,1,y);
    end
end

%---------------
% Creating Matrix (ClinOne)
ClinTwo = zeros(dNo,year);

% Loading results from Mat(:,1,:)
for y = 1:year
    for d = 1:dNo
        ClinTwo(d,y) = Mat(d,2,y);
    end
end

%---------------
% Creating Matrix (ClinOne)
ClinThree = zeros(dNo,year);

% Loading results from Mat(:,1,:)
for y = 1:year
    for d = 1:dNo
        ClinThree(d,y) = Mat(d,3,y);
    end
end

%-------------------------------------------------------------
% GRAPHIC DISPLAY OF RESULTS

% 2D SCATTERGRAM FOR ALL CLINICIANS IN FIRST YEAR OF PRACTICE
% A 2D scattergram is prepared where the number of cases seen for each
%     condition by each clinician in the first year of practice is
%     displayed, using color to indicate the number of cases for each
%     condition modelled

ListLength = StNo*dNo;
List = zeros(ListLength,3);

% Loading Clinician identity numbers into column 1 of List
for c=1:StNo
    for i = 1:dNo
        List ((dNo*(c-1))+i,1) = c;
    end
end

% Loading Disease identity numbers into column 2 of List
dgrp = ListLength/dNo;
for c = 1:dgrp
    for i = 1:dNo
        List ((dNo*(c-1))+i,2) = i;
    end
end

% Loading disease occurences into List
for s = 1:StNo
```

```
    for d=1:dNo
        % values for List location
        a = (s-1)*dNo;
        List(a+d,3)= Mat(d,s,1);
    end
end

% Shifting '0' results to 0 placements
for a=1:ListLength
    if List(a,3)==0
        List(a,1)=0;
        List(a,2)=0;
    end
end

% Defining Axes and Contents for Graph
Clinicians = List(:,1);
Conditions = List(:,2);
CasesPerYear = List(:,3);

% Defining marker size
s=10;

% Creating a matrix 'c' to receive RGB values
c = zeros (ListLength,3);

% Defining colors for results displayed in RGB Vectors
for i = 1:ListLength
    if  List(i,3) >= 300
        c(i,1) = 0.7;
        c(i,2) = 0;
        c(i,3) = 0.7;
    end

    if  List(i,3) >= 100 && List (i,3) < 300
        c(i,1) = 1;
        c(i,2) = 0;
        c(i,3) = 0;
    end

    if  List(i,3) >= 50 && List (i,3) < 100
        c(i,1) = 0;
        c(i,2) = 1;
        c(i,3) = 0;
    end

    if  List(i,3) >= 20 && List (i,3) < 50
        c(i,1) = 1;
        c(i,2) = 0.7;
        c(i,3) = 0;
    end

    if  List(i,3) >= 10 && List (i,3) < 20
        c(i,1) = 0;
        c(i,2) = 0;
        c(i,3) = 1;
    end

    if  List(i,3) >= 2 && List (i,3) < 10
```

```
        c(i,1) = 0.1;
        c(i,2) = 0.8;
        c(i,3) = 1;
    end

    if  List(i,3) == 1
        c(i,1) = 0;
        c(i,2) = 0;
        c(i,3) = 0;
    end
end

% Setting the font size for all graphs
set(0,'DefaultAxesFontSize',18);

subplot(2,3,1);
scatter(Clinicians,Conditions,s,c,'filled')


% Labelling the scattergram
title('1st Year All Clinicians')
ylabel('Condition Code')
xlabel('Clinicians')
hold

%------------------
% SINGLE COLUMN SCATTERGRAM SHOWING ALL CLINICIANS FOR WHOLE OF CAREERS
% A single column scattergram is prepared where the number of cases for
%     each condition for all clinicians across the entirety of their
%     careers is displayed, using color to indicate the number of cases
%     for each condition modelled

ListLength = dNo;
List = zeros(ListLength,3);

% Loading Disease identity numbers into column 1 of List
for d = 1:dNo
    List(d,1) = d;
end

% Loading disease occurences into List
for t = 1:dNo

    List(t,2)= TotAllClin(t,year+1);
end

% Loading scattergram column position into List
for p = 1:dNo

    List(p,3)= 1;
end

% Defining Axes and Contents for Graph
Condition = List(:,1);
Cases = List(:,2);
Position = List(:,3);

% Defining marker size
```

```
s=20;

% Creating a matrix 'c' to receive RGB values
c = zeros (ListLength,3);

% Defining colors for results displayed in RGB Vectors
for i = 1:ListLength
    if  List(i,2) >= 300
        c(i,1) = 0.7;
        c(i,2) = 0;
        c(i,3) = 0.7;
    end

    if  List(i,2) >= 100 && List (i,2) < 300
        c(i,1) = 1;
        c(i,2) = 0;
        c(i,3) = 0;
    end

    if  List(i,2) >= 50 && List (i,2) < 100
        c(i,1) = 0;
        c(i,2) = 1;
        c(i,3) = 0;
    end

    if  List(i,2) >= 20 && List (i,2) < 50
        c(i,1) = 1;
        c(i,2) = 0.7;
        c(i,3) = 0;
    end

    if  List(i,2) >= 10 && List (i,2) < 20
        c(i,1) = 0;
        c(i,2) = 0;
        c(i,3) = 1;
    end

    if  List(i,2) >= 2 && List (i,2) < 10
        c(i,1) = 0.1;
        c(i,2) = 0.8;
        c(i,3) = 1;
    end

    if  List(i,2) == 1
        c(i,1) = 0;
        c(i,2) = 0;
        c(i,3) = 0;
    end
end

% Setting the font size for all graphs
set(0,'DefaultAxesFontSize',18);

subplot(2,3,3);
scatter(Position,Condition,s,c,'filled')

% Constructing the legend
% Defining RGB values for each 'box' - 'Patch'
cmap = [0 0 0; 0.1 0.8 1; 0 0 1; 1 0.7 0; 0 1 0; 1 0 0; 0.7 0 0.7];
```

```
% Labelling each colored box
lbl = {'1','2-9','10-19','20-49','50-99','100-299','>= 300'};
% Distributing and placing labels and boxes
for ii=1:size(cmap,1)
    p(ii)=patch(NaN,NaN,cmap(ii,:));
end
legend(p,lbl,'Location','EastOutside');

% Labelling the scattergram
title('All Clinicians & Years')
ylabel('Condition Code')
xlabel('Cases')
hold


%----------
% 2D SCATTERGRAM OF CLINICIAN 'NUMBER ONE' ENTIRE CAREER
% Three 2D scattergrams are prepared where the number of cases for
%     each condition for the first three clinicians for each year of
%     their careers is displayed, using color to indicate the number of
%     cases for each condition modelled

ListLength = year*dNo;
List = zeros(ListLength,3);

% Loading Year identity numbers into column 1 of List
for c=1:year
    for i = 1:dNo
        List ((dNo*(c-1))+i,1) = c;
    end
end

% Loading Disease identity numbers into column 2 of List
for y = 1:year
    for i = 1:dNo
        List ((dNo*(y-1))+i,2) = i;
    end
end

% Loading disease occurences into List
for y = 1:year
    for d=1:dNo
        a = (y-1)*dNo;
        % values for List location
        List(a+d,3)= Mat(d,1,y);
    end
end

% Shifting '0' results to 0 placements
for a=1:ListLength

    if List(a,3)==0
        List(a,1)=0;
        List(a,2)=0;
    end
end

% Defining Axes and Contents for Graph
```

```
Year = List(:,1);
Conditions = List(:,2);
CasesPerYear = List(:,3);

% Defining marker size
s=20;

% Creating a matrix 'c' to receive RGB values
c = zeros (ListLength,3);

% Defining colors for results displayed in RGB Vectors
for i = 1:ListLength
    if  List(i,3) >= 300
        c(i,1) = 0.7;
        c(i,2) = 0;
        c(i,3) = 0.7;
    end

    if  List(i,3) >= 100 && List (i,3) < 300
        c(i,1) = 1;
        c(i,2) = 0;
        c(i,3) = 0;
    end

    if  List(i,3) >= 50 && List (i,3) < 100
        c(i,1) = 0;
        c(i,2) = 1;
        c(i,3) = 0;
    end

    if  List(i,3) >= 20 && List (i,3) < 50
        c(i,1) = 1;
        c(i,2) = 0.7;
        c(i,3) = 0;
    end

    if  List(i,3) >= 10 && List (i,3) < 20
        c(i,1) = 0;
        c(i,2) = 0;
        c(i,3) = 1;
    end

    if  List(i,3) >= 2 && List (i,3) < 10
        c(i,1) = 0.1;
        c(i,2) = 0.8;
        c(i,3) = 1;
    end

    if  List(i,3) == 1
        c(i,1) = 0;
        c(i,2) = 0;
        c(i,3) = 0;
    end
end

% Setting the font size for all graphs
set(0,'DefaultAxesFontSize',18);

subplot(2,3,4);
```

```
scatter(Year,Conditions,s,c,'filled')

% Labelling the scattergram
title('Clinician One All Years')
ylabel('Condition Code')
xlabel('Year')
hold

%------------------
% 2D SCATTERGRAM OF CLINICIAN 'NUMBER TWO' ENTIRE CAREER
ListLength = year*dNo;
List = zeros(ListLength,3);

% Loading Year identity numbers into column 1 of List
for c=1:year
    for i = 1:dNo
        List ((dNo*(c-1))+i,1) = c;
    end
end

% Loading Disease identity numbers into column 2 of List
for y = 1:year
    for i = 1:dNo
        List ((dNo*(y-1))+i,2) = i;
    end
end

% Loading disease occurences into List
for y = 1:year
    for d=1:dNo
        a = (y-1)*dNo;
        % values for List location
        List(a+d,3)= Mat(d,2,y);
    end
end

% Shifting '0' results to 0 placements
for a=1:ListLength

    if List(a,3)==0
        List(a,1)=0;
        List(a,2)=0;
    end
end

% Defining Axes and Contents for Graph
Year = List(:,1);
Conditions = List(:,2);
CasesPerYear = List(:,3);

% Defining marker size
s=20;

% Creating a matrix 'c' to receive RGB values
c = zeros (ListLength,3);

% Defining colors for results displayed in RGB Vectors
for i = 1:ListLength
    if  List(i,3) >= 300
```

```
        c(i,1) = 0.7;
        c(i,2) = 0;
        c(i,3) = 0.7;
    end

    if  List(i,3) >= 100 && List (i,3) < 300
        c(i,1) = 1;
        c(i,2) = 0;
        c(i,3) = 0;
    end

    if  List(i,3) >= 50 && List (i,3) < 100
        c(i,1) = 0;
        c(i,2) = 1;
        c(i,3) = 0;
    end

    if  List(i,3) >= 20 && List (i,3) < 50
        c(i,1) = 1;
        c(i,2) = 0.7;
        c(i,3) = 0;
    end

    if  List(i,3) >= 10 && List (i,3) < 20
        c(i,1) = 0;
        c(i,2) = 0;
        c(i,3) = 1;
    end

    if  List(i,3) >= 2 && List (i,3) < 10
        c(i,1) = 0.1;
        c(i,2) = 0.8;
        c(i,3) = 1;
    end

    if  List(i,3) == 1
        c(i,1) = 0;
        c(i,2) = 0;
        c(i,3) = 0;
    end
end

% Setting the font size for all graphs
set(0,'DefaultAxesFontSize',18);

subplot(2,3,5);
scatter(Year,Conditions,s,c,'filled')

% Labelling the scattergram
title('Clinician Two All Years')
ylabel('Condition Code')
xlabel('Year')
hold

%------------------
% 2D SCATTERGRAM OF CLINICIAN 'NUMBER THREE' ENTIRE CAREER
ListLength = year*dNo;
List = zeros(ListLength,3);
```

```
% Loading Year identity numbers into column 1 of List
for c=1:year
    for i = 1:dNo
        List ((dNo*(c-1))+i,1) = c;
    end
end

% Loading Disease identity numbers into column 2 of List
for y = 1:year
    for i = 1:dNo
        List ((dNo*(y-1))+i,2) = i;
    end
end

% Loading disease occurences into List
for y = 1:year
    for d=1:dNo
        a = (y-1)*dNo;
        % values for List location
        List(a+d,3)= Mat(d,3,y);
    end
end

% Shifting '0' results to 0 placements
for a=1:ListLength

    if List(a,3)==0
        List(a,1)=0;
        List(a,2)=0;
    end
end

% Defining Axes and Contents for Graph
Year = List(:,1);
Conditions = List(:,2);
CasesPerYear = List(:,3);

% Defining marker size
s=20;

% Creating a matrix 'c' to receive RGB values
c = zeros (ListLength,3);

% Defining colors for results displayed in RGB Vectors
for i = 1:ListLength
    if  List(i,3) >= 300
        c(i,1) = 0.7;
        c(i,2) = 0;
        c(i,3) = 0.7;
    end

    if  List(i,3) >= 100 && List (i,3) < 300
        c(i,1) = 1;
        c(i,2) = 0;
        c(i,3) = 0;
    end

    if  List(i,3) >= 50 && List (i,3) < 100
        c(i,1) = 0;
```

```
        c(i,2) = 1;
        c(i,3) = 0;
    end

    if  List(i,3) >= 20 && List (i,3) < 50
        c(i,1) = 1;
        c(i,2) = 0.7;
        c(i,3) = 0;
    end

    if  List(i,3) >= 10 && List (i,3) < 20
        c(i,1) = 0;
        c(i,2) = 0;
        c(i,3) = 1;
    end

    if  List(i,3) >= 2 && List (i,3) < 10
        c(i,1) = 0.1;
        c(i,2) = 0.8;
        c(i,3) = 1;
    end

    if  List(i,3) == 1
        c(i,1) = 0;
        c(i,2) = 0;
        c(i,3) = 0;
    end
end

% Setting the font size for all graphs
set(0,'DefaultAxesFontSize',18);

subplot(2,3,6);
scatter(Year,Conditions,s,c,'filled')

% Labelling the scattergram
title('Clinician Two All Years')
ylabel('Condition Code')
xlabel('Year')
hold


%------------------
% 3D SCATTERGRAM
% A 3D scattergram is prepared where the number of cases seen for each
%     condition by each clinician in each year of practice is
%     displayed, using color to indicate the number of cases for each
%     condition modelled

% Constructing a matrix to receive values for 3D graph
ListLength = StNo*dNo*year;
List = zeros(ListLength,4);

% Loading Clinician identity numbers into column 1 of List
ClinVal = year*dNo;

for c=1:StNo
    for i = 1:ClinVal
        List ((ClinVal*(c-1))+i,1) = c;
```

```
    end
end

% Loading Disease identity numbers into column 2 of List
dgrp = ListLength/dNo;

for c = 1:dgrp
    for i = 1:dNo
        List ((dNo*(c-1))+i,2) = i;
    end
end

% Loading Year identity numbers into column 3 of List
for s = 1:StNo
    for y = 1:year
        for d=1:dNo
            a = (s-1)*year*dNo;
            b = (y-1)*dNo;
            List(a+b+d,3)=y;
        end
    end
end

% Loading disease occurences into List
for s = 1:StNo
    for y = 1:year
        for d=1:dNo
            % values for List location
            a = (s-1)*year*dNo;
            b = (y-1)*dNo;
            List(a+b+d,4)= Mat(d,s,y);
        end
    end
end

% Shifting '0' results to 0 placements
for a=1:ListLength

    if List(a,4)==0
        List(a,1)=0;
        List(a,2)=0;
        List(a,3)=0;
        List(a,4)=0;
    end
end

% Defining Axes and Contents for Graph
Clinicians = List(:,1);
Conditions = List(:,2);
Years = List(:,3);
CasesPerYear = List(:,4);

% Defining size of marker
s=3.5;
% Creating a matrix 'c' to receive RGB values
c = zeros (ListLength,3);

% Defining colors for results displayed in RGB Vectors
for i = 1:ListLength
```

```
    if  List(i,4) >= 300
        c(i,1) = 0.7;
        c(i,2) = 0;
        c(i,3) = 0.7;
    end

    if  List(i,4) >= 100 && List (i,4) < 300
        c(i,1) = 1;
        c(i,2) = 0;
        c(i,3) = 0;
    end

    if  List(i,4) >= 50 && List (i,4) < 100
        c(i,1) = 0;
        c(i,2) = 1;
        c(i,3) = 0;
    end

    if  List(i,4) >= 20 && List (i,4) < 50
        c(i,1) = 1;
        c(i,2) = 0.7;
        c(i,3) = 0;
    end

    if  List(i,4) >= 10 && List (i,4) < 20
        c(i,1) = 0;
        c(i,2) = 0;
        c(i,3) = 1;
    end

    if  List(i,4) >= 2 && List (i,4) < 10
        c(i,1) = 0.1;
        c(i,2) = 0.8;
        c(i,3) = 1;
    end


    if  List(i,4) == 1
        c(i,1) = 0;
        c(i,2) = 0;
        c(i,3) = 0;
    end
end

% Plotting the scattergram
subplot(2,3,2);
scatter3(Years,Clinicians,Conditions,s,c,'filled')
view(-34,20)

% Labelling the scattergram
title('All Years & Clinicians')
xlabel('Years')
ylabel('Clinicians')
zlabel('Condition Code')

%  Clearing variables

clear a AllPtPrSt AtRepPts Atrit b bx c Cases CasesPerYear ChJMax Clin
```

```
clear Clinicians ClinVal cmap cnt Col Conditions CpY d dgrp di dNo e f g
clear i ii Jobs lbl List ListLength Mat max min NewPracPts NewPtsYr NPY
clear p Page Prev PtsYr r Recip rem Row s st StNo t TotPts var x y year
clear Year Years ys Condition Position

% END SCRIPT
```
